# Classification of Agarwood Oil Using an Electronic Nose

**DOI:** 10.3390/s100504675

**Published:** 2010-05-06

**Authors:** Wahyu Hidayat, Ali Yeon Md. Shakaff, Mohd Noor Ahmad, Abdul Hamid Adom

**Affiliations:** Sensor Technology and Applications Research Cluster, Universiti Malaysia Perlis (UniMAP), 01000 Kangar, Perlis, Malaysia; E-Mails: aliyeon@unimap.edu.my (A.Y.M.S.); mohdnoor@unimap.edu.my (M.N.A.); abdhamid@unimap.edu.my (A.H.A.)

**Keywords:** agarwood oil, e-nose, HCA, PCA, ANN, dimensionality reduction

## Abstract

Presently, the quality assurance of agarwood oil is performed by sensory panels which has significant drawbacks in terms of objectivity and repeatability. In this paper, it is shown how an electronic nose (e-nose) may be successfully utilised for the classification of agarwood oil. Hierarchical Cluster Analysis (HCA) and Principal Component Analysis (PCA), were used to classify different types of oil. The HCA produced a dendrogram showing the separation of e-nose data into three different groups of oils. The PCA scatter plot revealed a distinct separation between the three groups. An Artificial Neural Network (ANN) was used for a better prediction of unknown samples.

## 1. Introduction

Agarwood is the well-known name for a resinous heartwood from ‘wounded/infected’ Aquilaria trees, a tropical forest product which has a high value in international trading. There are increasing demands for agarwood-based products for use in medicine, perfume, and incense. Agarwood is traded in the form of product derivatives such as wood chips, powder, and oil. The wholesale price for high quality agarwood oils is around US$30,000–US$50,000 per liter [1], depending on the oil quality, which is based upon the fragrance strength and longevity, resin content, geographical origin, and oil purity [2].

Traditionally, agarwood grading has been performed by trained human graders (sensory panels). However, the method has disadvantages in terms of objectivity and repeatability [3]. In addition, a human nose cannot tolerate a high number of samples because it fatigues rapidly with increasing number of samples. In this paper, it is shown how an electronic nose (e-nose) may be used to resolve these issues. A commercial e-nose, the Cyranose 320 (Smith Detection, USA) was used to collect the smell (fragrance) data (herein termed as ‘smellprint’) which was then processed on a personal computer using different pattern recognition methods: Hierarchical Cluster Analysis (HCA), Principal Component Analysis (PCA) and Artificial Neural Network (ANN).

## 2. Experimental

**Figure 1.**
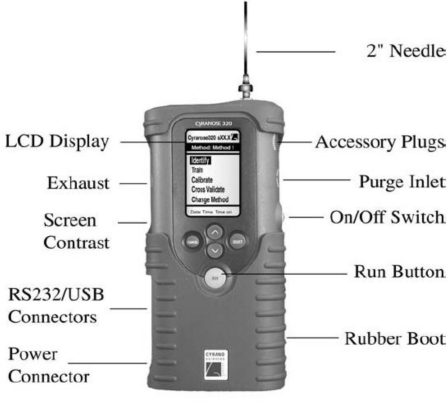
The Cyranose 320 [4].

An experiment aimed to produce a data set with robust consistency was conducted using a Cyranose 320 (see Figure 1). The important features of the Cyranose 320 are provided in Table 1 [4]. The acquired raw data was then processed and interpreted into meaningful information. The samples were obtained from three groups of agarwood oils originating from Laos, Johor (Malaysia) and Terengganu (Malaysia), which were labelled as G12, G22, and G32, respectively. Two μL samples from each locality was diluted using 500 mL glycerol as a solvent. The samples were placed in a 40 mL stoppered vial connected to a Cyranose 320 via an inert tubing and heated to 60 °C in a heater block. Each experiment was run for 30 min.

**Figure 2.**
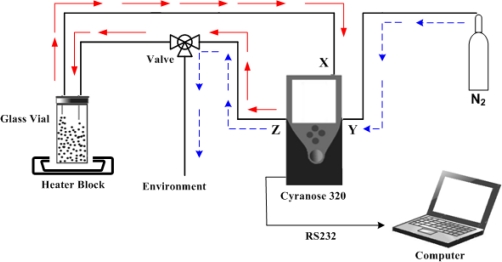
Experimental setup for the classification of agarwood oil.

Figure 2 illustrates an airtight recycle system for continuous sniffing. This approach could avoid volatile concentration loss and also pressure loss at the glass vial, which would affect the experiment. An automatic valve as well as a small pump was provided in the Cyranose 320 to control the system flow. In addition, the use of an extra valve mechanism outside the Cyranose 320 was improvised to facilitate sensor cleansing. In Figure 2, the red arrows (solid-line) indicate a sampling cycle. An internal valve was switched to the sample inlet (X). The volatile was then sucked into the e-nose through inlet (X) and was retained for 20 s in the sensor chamber before being removed through outlet (Z). At the same time, the external valve allowed volatile from Z to fill the glass vial. At the end of 10 cycles, the internal valve allowed the nitrogen gas (N2) to purge the sensors via inlet (Y) and removed out to the atmosphere through port (Z). The purge cycle is illustrated by the blue arrows (dashed-line) in Figure 2. The experiments were controlled by the Cyranose 320 according to the set-up parameters as shown in Table 2.

## 3. Results and Discussion

### 3.1. Smellprint

The agarwood oil volatiles are adsorbed on the sensor’s surfaces and cause a change in its resistance. The response of the sensor is defined by using fractional baseline manipulation [5]:

(1) $$\frac{{\Delta R}_{s}}{R_{s,0}}=\frac{R_{s,n}-R_{s,0}}{R_{s,0}}$$

where ΔR_s_ is the resistance change of sensor s, *R_s,n_* is the output resistance and *R_s,0_* is the baseline output. The subscript index s is the sensor number used in the Cyranose (s = 1…32) and n is an index for the number of data (n = 1…N).

**Figure 3.**
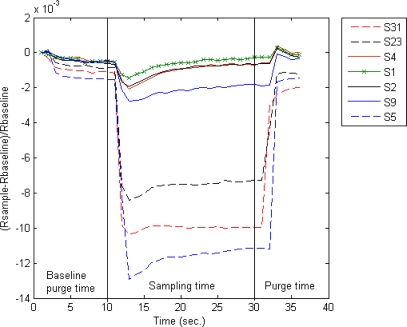
Measurements taken from seven of the sensors for one sampling cycle.

As an example, Figure 3 shows responses from seven sensors of the Cyranose 320. The data was taken from one sampling cycle of a G12 experiment. The figure also illustrates the base line purge time, sampling time and purge time.

**Figure 4.**
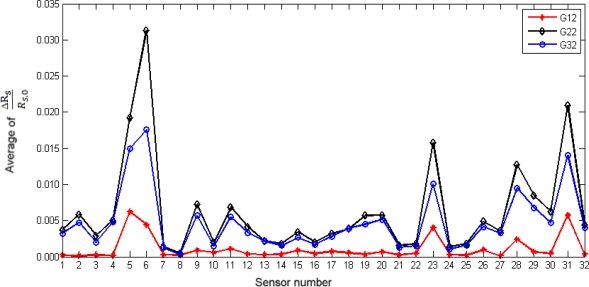
Smellprints of three different agarwood oils.

The average of values evaluated by Equation (1) is plotted as shown in Figure 4, and corresponds to the smellprints of the three different agarwood oils. Sensors with high responses are analyzed by comparing their peaks and profiles [6]. Sensor numbers 6, 31, 5, 23, and 28 (in the order of diminishing responses) have higher responses compared to the rest when exposed to the volatiles of the different grade of oils. However, the analysis of smellprints becomes more difficult when there is an increase in the number of samples having overlapping profiles. This issue can be solved using graphical methods based on statistical theories [7], and this was adopted and presented in the next section.

### 3.2. Statistical Analysis

There are many statistical-based methods for processing e-nose data. This paper presents the implementation of the Hierarchical Cluster Analysis (HCA) and Principal Component Analysis (PCA) to distinguish the different agarwood oil grades.

#### 3.2.1. Hierarchical Cluster Analysis (HCA)

The aim of performing Hierarchical cluster analysis (HCA) is to separate data into specific groups by considering similarity criterion, a distance metric such as Euclidean distance, as follows:

(2) $$d_{\mathrm{ij}}={\left(\sum_{k=1}^{K}{\left(r_{\mathrm{ik}}-r_{\mathrm{jk}}\right)}^{2}\right)}^{1/2}$$

where K is the number of variables (in this case K is equal to 32 that is the number of sensors in the Cyranose), while *i* and *j* are the indices for groups of samples. Hence, a parameter to measure the level of similarity, *S_ij_*, is defined as [8]:

(3) $$S_{\mathrm{ij}}=1-d_{\mathrm{ij}}/\text{max}\left\{d_{\mathrm{ij}}\right\}$$

**Figure 5.**
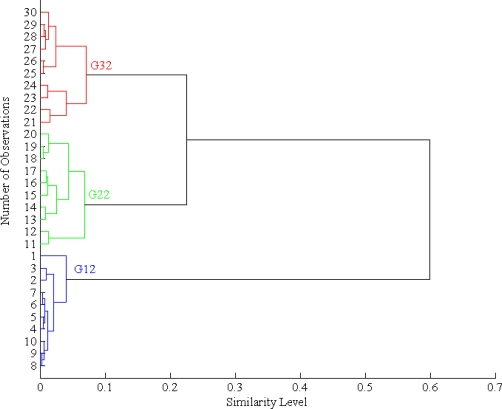
A dendrogram for the three-object data set from each 10 samples of G12, G22, and G32.

The computational process of *S_ij_* using MATLAB gives a dendrogram as shown in Figure 5. The figure proved the capability of HCA to differentiate between G12, G22, and G33.

#### 3.2.2. Principal Component Analysis (PCA)

**Figure 6.**
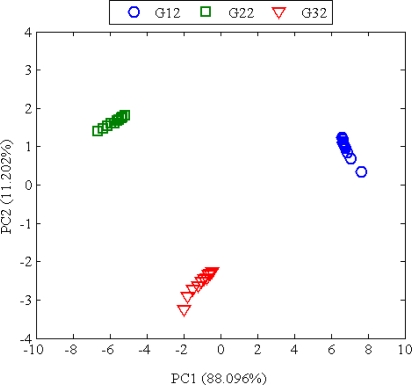
Principal components score plot proves the capability of e-nose to classify the different types of oils G12, G22, and G32.

Principal component analysis (PCA) is an unsupervised statistical method that generates a new set of variables, called principal components. Each principal component is a linear combination of the original variables (*r_s,n_*) defined by [9]:

(4) $${\mathrm{PC}}_{p,n}=\alpha_{p,1}\gamma_{1,n}+\alpha_{p,2}\gamma_{2,n}+\cdots +\alpha_{p,s}\gamma_{s,n}$$

where *PC_p,n_* is the notation for the *p*-th order principal component for the overall n number of data and is termed as *scores*. Coefficients transformations (*α_p,s_*), referred as *loadings,* are obtained by taking elements of the eigenvectors from the covariant of the original data. The eigenvalue represents the variance associated with each principal component. By using MATLAB software, the two principal components {*PC*_1,*n*_, *PC*_2,*n*_} are obtained and have the two greatest variances: 88.096% and 11.202% (or total cumulative variance of 99.298%). The results of the PCA analysis are shown in Figure 6. The scores of the three groups of oils are plotted for principal component 2 (PC2) *versus* principal component 1 (PC1). The discrimination between the different types of oils can be clearly seen from the figure.

### 3.3. Artificial Neural Network (ANN)

#### 3.3.1. Result from 32 Sensors as Input

The previous two statistical approaches, HCA and PCA, successfully showed their capabilities to distinguish different types of agarwood oils. Both are typically used for exploratory data analysis to see how the multivariate data is clustered and to assess the linear separability of the odour classes. However, in cases where prediction is required (e.g., when implementing an automated classifier), the ANN is the more appropriate tool [7]. This section presents the use of ANN as an alternative choice to solve the classification problem in this work.

**Figure 7.**
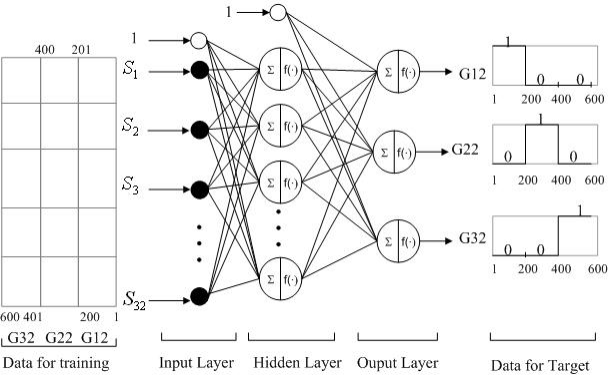
The architecture of three layers ANN with Levenberg-Marquardt algorithm applied for training and identification G12, G22, and G32.

In this experiment, the backpropagation ANN with the Levenberg-Marquardt training algorithm was applied. The training used all the 32 sensors as inputs, 20 neurons in the single hidden layer, and three neurons at the output layer. The activation functions used are sigmoid and identity functions at the hidden and output layer, respectively. Figure 7 illustrates the structure of the ANN and how the input and output data were organised and indexed.

The same experimental procedure was carried out for the training as well as the testing data. The 200 raw data points were collected by experiments for each of the oil types G12, G22, and G32. This resulted in 600 data points for use in training and validation. After performing baseline manipulation and auto-scaling, the data were organised in one matrix to be fed as input. The testing data was collected on a different day, by sniffing the odour of the oils in nine vials that were assigned for testing only. The total of 1,800 data points was used as the testing data.

As shown in Table 3, the ANN performed very well in discriminating the three types of oils with 5.713345 × 10^−8^ mean square error (MSE) and 100% prediction performance. The prediction performance was defined as: (number of correct classification/number of total data) × 100%.

#### 3.3.2. Results from Selected Sensors

The use of too many sensors may increase noise, redundant information and provides no real benefit, whereas minimizing the number of sensors can result in the loss of some useful input information [10,11]. Thus, optimization for a specific application can be achieved by observing the sensors which provide high contribution to the system and eliminating the lower ones.

In this work, PCA is used to reduce high dimensionality data and to improve ANN training [12]. Table 4 is the list of loadings for PC1 that was sorted in descending order. The summation of the correlation coefficient from the matrix response data for each sensor is also provided in Table 4 as a comparison with the PCA results. It is evident that the higher loading values of PC1 for all sensors correspond to the less correlated sensors.

From Table 4, the data from the five least correlated sensors (sensor number 23, 31, 1, 2, and 4) are selected as input for the ANN training. The result shows an improvement where the ANN has a lower MSE, 8.20279 × 10^−9^ and 100% successful prediction of unknown data. The time spent for training and identification also decrease. For specific application, in this case to discriminate the three different types of agarwood oils G12, G22, and G32, the five selected sensors (from the 32 total sensors in the Cyranose) is an effective choice in terms of speed of detection and high accuracy. Attempts to further reduce the number of sensors was not successful. Table 5 compares the results of the ANN training for the case of using reduced number of sensors.

## 4. Conclusions

Classification of agarwood oils using an e-nose is able to provide rapid and accurate results. The data from the Cyranose 320 were processed using in-house developed software in MATLAB and was able to identify three different types of agarwood oils G12, G22, and G32. Hierarchical cluster analysis (HCA) and principal component analysis (PCA) were successful in separating the samples into different groups or clusters. ANN was also successfully applied to predict unknown agarwood samples. The optimum number of sensors for this application has been determined by PCA analysis, which subsequently minimize the number of ANN input variables. The current research in our laboratories is to verify the purity and grading of the oil based on quantitative analysis using the e-nose.

## Figures and Tables

**Table 1.** Important features of the Cyranose 320 [4].

Sensors	32 polymer carbon black composites
Operating Temperature	0 to 40 °C (32 to 104 °F)
Response Time	10 sec
Sampling Pump	Low: 50 mL/min, Medium: 120 mL/min,
	High: 180 mL/min.
Communication	RS–232 @ 9,600 to 57,600 bps
Algorithms	PCA, KNN, K-means, CDA

**Table 2.** Cyranose 320 parameter set up for sampling agarwood oil.

	**Run time**	**Pump speed**

Baseline purge time	10 sec	120 mL/min

Sampling time		
Draw 1	20 sec	180 mL/min

Purge time		
1st air intake purge	5 sec	180 mL/min
2nd sample gas purge	30 sec	180 mL/min

Digital filtering	On	

Substrate heater temperature	42 °C	

Training repeat count	10	

**Table 3.** ANN output for 32 sensors.

**No.**	**Sensor selection**	**MSE**	**Gradient**	**Sample**	**Target**			**ANN Output (Averaged)**			**Accuracy**
					**T1**	**T2**	**T3**	**O1**	**O2**	**O3**	
1	All	5.7133 × 10^−8^	0.3536	G12	1	0	0	0.9999	0.0001	0.0000	100%
				G22	0	1	0	0.0003	0.9997	0.0000	100%
				G32	0	0	1	0.0005	0.0002	0.9997	100%

**Table 4.** The value of total correlation coefficient and loadings of PC1 for seven sensors.

**No**	**Sensor number**	**The summation of correlation coefficient**	**Sensor number**	**Loadings for PC1**
1	23	5.4960	23	−0.03041
2	31	16.016	31	−0.09815
3	1	19.334	1	−0.12595
4	2	24.291	2	−0.15748
5	4	25.343	4	−0.16391
6	9	25.714	9	−0.16607
7	5	26.599	5	−0.17615

**Table 5.** The comparison performance of ANN using selected sensors.

**No.**	**Sensor selection**	**MSE**	**Gradient**	**Sample**	**Target**			**ANN Output (Averaged)**			**Accuracy**	

					T1	T2	T3	O1	O2	O3		
1	S23, S31, S1, S2, S4	8.2038 × 10^−9^	0.1074	G12	1	0	0	0.9983	0.0045	0.0070	100%	
				G22	0	1	0	0.0001	0.9999	0.0070	100%	
				G32	0	0	1	0.0001	0.0000	0.9999	100%	

2	S23 and S31	8.9927 × 10^−8^	0.00246	G12	1	0	0	0.9803	0.0125	0.0070		100%
				G22	0	1	0	0.0010	0.9999	0.0009		100%
				G32	0	0	1	0.6203	0.0000	0.3797		37.18%
